# New prognostic system based on inflammation and liver function predicts prognosis in patients with advanced unresectable hepatocellular carcinoma treated with atezolizumab plus bevacizumab: A validation study

**DOI:** 10.1002/cam4.5495

**Published:** 2022-12-09

**Authors:** Toshifumi Tada, Takashi Kumada, Atsushi Hiraoka, Kazuya Kariyama, Joji Tani, Masashi Hirooka, Koichi Takaguchi, Masanori Atsukawa, Shinya Fukunishi, Ei Itobayashi, Kunihiko Tsuji, Kazuto Tajiri, Hironori Ochi, Toru Ishikawa, Satoshi Yasuda, Chikara Ogawa, Hidenori Toyoda, Takeshi Hatanaka, Takashi Nishimura, Satoru Kakizaki, Kazuhito Kawata, Noritomo Shimada, Fujimasa Tada, Kazuhiro Nouso, Akemi Tsutsui, Hideko Ohama, Asahiro Morishita, Takuya Nagano, Norio Itokawa, Tomomi Okubo, Taeang Arai, Hisashi Kosaka, Michitaka Imai, Atsushi Naganuma, Shinichiro Nakamura, Yohei Koizumi, Masaki Kaibori, Hiroko Iijima, Yoichi Hiasa, the Real‐life Practice Experts for HCC (RELPEC) Study Group and the Hepatocellular Carcinoma Experts from 48 clinics in Japan (HCC 48) Group

**Affiliations:** ^1^ Department of Internal medicine, Division of Gastroenterology and Hepatology Hyogo Medical University Hyogo Japan; ^2^ Department of Internal Medicine Japanese Red Cross Himeji Hospital Hygo Japan; ^3^ Department of Nursing Gifu Kyoritsu University Gifu Japan; ^4^ Gastroenterology Center Ehime Prefectural Central Hospital Ehime Japan; ^5^ Department of Gastroenterology Okayama City Hospital Okayama Japan; ^6^ Department of Gastroenterology and Hepatology Kagawa University Kagawa Japan; ^7^ Department of Gastroenterology and Metabology Ehime University Graduate School of Medicine Ehime Japan; ^8^ Department of Hepatology Kagawa Prefectural Central Hospital Kagawa Japan; ^9^ Division of Gastroenterology and Hepatology, Department of Internal Medicine Nippon Medical School Tokyo Japan; ^10^ Department of Gastroenterology Osaka Medical and Pharmaceutical University Osaka Japan; ^11^ Department of Gastroenterology Asahi General Hospital Chiba Japan; ^12^ Center of Gastroenterology Teine Keijinkai Hospital Hokkaido Japan; ^13^ Department of Gastroenterology Toyama University Hospital Toyama Japan; ^14^ Hepato‐biliary Center Japanese Red Cross Matsuyama Hospital Ehime Japan; ^15^ Department of Gastroenterology Saiseikai Niigata Hospital Niigata Japan; ^16^ Department of Gastroenterology and Hepatology Ogaki Municipal Hospital Gifu Japan; ^17^ Department of Gastroenterology Japanese Red Cross Takamatsu Hospital Kagawa Japan; ^18^ Department of Gastroenterology Gunma Saiseikai Maebashi Hospital Gunma Japan; ^19^ Department of Clinical Research National Hospital Organization Takasaki General Medical Center Gunma Japan; ^20^ Department of Hepatology Hamamatsu University School of Medicine Shizuoka Japan; ^21^ Division of Gastroenterology and Hepatology Otakanomori Hospital Chiba Japan; ^22^ Department of Surgery Kansai Medical University Osaka Japan; ^23^ Department of Gastroenterology National Hospital Organization Takasaki General Medical Center Gunma Japan

**Keywords:** atezolizumab plus bevacizumab, Glasgow prognostic score, hepatocellular carcinoma, neo‐Glasgow prognostic score, survival

## Abstract

**Aim:**

Recently, the neo‐Glasgow prognostic score (GPS), a composite biomarker determined by the C‐reactive protein level and albumin–bilirubin grade, was developed to predict outcomes in hepatocellular carcinoma (HCC) patients who undergo hepatic resection. The present research investigated whether the neo‐GPS could predict prognosis in HCC patients treated with atezolizumab plus bevacizumab (Atez/Bev).

**Methods:**

A total of 421 patients with HCC who were treated with Atez/Bev were investigated.

**Results:**

Multivariate Cox hazards analysis showed that a GPS of 1 (hazard ratio (HR), 1.711; 95% confidence interval (CI), 1.106–2.646) and a GPS of 2 (HR, 4.643; 95% CI, 2.778–7.762) were independently associated with overall survival. Conversely, multivariate Cox hazards analysis showed that a neo‐GPS of 1 (HR, 3.038; 95% CI, 1.715–5.383) and a neo‐GPS of 2 (HR, 5.312; 95% CI, 2.853–9.890) were also independently associated with overall survival in this cohort. Additionally, cumulative overall survival rates differed significantly by GPS and neo‐GPS (*p* < 0.001). The neo‐GPS, compared with the GPS, had a lower Akaike information criterion (1207 vs. 1,211, respectively) and a higher c‐index (0.677 vs. 0.652, respectively) regarding to overall survival. In a subgroup analysis of patients considered to have a good prognosis as confirmed using a Child–Pugh score of 5 (*p* = 0.001), a neutrophil‐to‐lymphocyte ratio <3 (*p* = 0.001), or an α‐fetoprotein level < 100 ng/mL (*p* < 0.001), those with a high neo‐GPS (≥1) had a statistically poorer overall survival than those with a low neo‐GPS.

**Conclusions:**

The neo‐GPS can predict prognosis in advanced unresectable HCC patients treated with Atez/Bev.

## Graphical abstract

The neo‐Glasgow prognostic score, based on C‐reactive protein level and albumin‐bilirubin grade, was developed as a new biomarker for hepatocellular carcinoma. High neo‐Glasgow prognostic scores were associated with poor outcomes in patients treated with atezolizumab plus bevacizumab.In comparison with the Glasgow prognostic score, the neo‐Glasgow prognostic score was associated with a lower Akaike information criterion value and higher c‐index for overall survival.

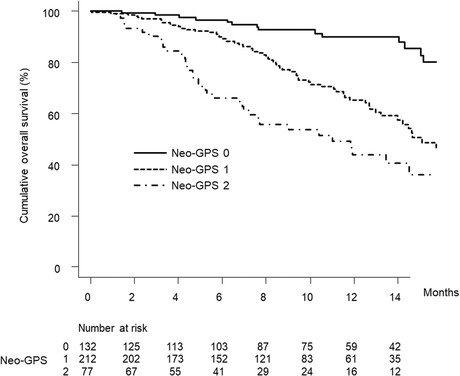

**Abbreviations**
AIC: Akaike information criterion
ALBI: albumin–bilirubin grade
Atez/Bev: atezolizumab plus bevacizumab
BCLC: Barcelona Clinic Liver Cancer
CI: confidence interval
CR: complete response
CRP: C‐reactive protein
CT: computed tomography
DCR: disease control rate
ECOG‐PS: Eastern Cooperative Oncology Group performance status
GPS: Glasgow prognostic score
HCC: hepatocellular carcinoma
HR: hazard ratio
NA: not available
NLR: neutrophil‐to‐lymphocyte ratio
PD: progressive disease
PR: partial response
SD: stable disease

## 1 INTRODUCTION

In 2020, primary hepatic malignancy was the sixth most generally confirmed malignant tumor and the third principal cause of malignant disease‐related mortality in the world, with about 906,000 newly confirms and 830,000 deaths, respectively.^1^ Hepatocellular carcinoma (HCC) consists about 80% of primary hepatic cancers and causes an important health issue in the world.^1^ Radical treatment for early‐stage HCC includes hepatectomy, transplantation, and local ablation therapy.^2^ Majority of patients for whom radical treatment is not recommended receive palliative treatments such as transarterial chemoembolization or systematic therapy.^2^

Atezolizumab plus bevacizumab (Atez/Bev) was recently confirmed as first‐choice systemic treatment for patients with advanced unresectable HCC. This new systemic treatment is composed of the combination of an immune checkpoint inhibitor (Atez) and an antibody against vascular endothelial growth factor (Bev).^3^ It is confirmed to have better therapeutic value, including improved outcomes in patients with HCC, than previous first‐choice systemic therapies involving tyrosine kinase inhibitors (i.e., lenvatinib and sorafenib).^3^, ^4^

Numerous indicators involving age, gender, nutritional status, HCC stage, level of α‐fetoprotein, and Eastern Cooperative Oncology Group performance status (ECOG‐PS) have been investigated as predictors of prognosis for patients who received HCC treatment.^5^, ^6^, ^7^, ^8^ Liver reserve, which can be evaluated using tools such as the albumin–bilirubin (ALBI) score/grade, is one of the most important indicators confirming the prognosis in patients with HCC.^9^ However, there are insufficient survival biomarkers, especially those that take liver function into account, in patients with unresectable HCC who received Atez/Bev therapy.

The existence of a systemic inflammatory reaction is related to unpreferable prognosis in patients with many kinds of cancers. Some recent researches have investigated that the existence of systemic inflammatory reaction and nutritional status predict cancer‐specific outcomes, including HCC.^10^, ^11^, ^12^, ^13^, ^14^, ^15^ These two factors can be assessed using the Glasgow prognostic score (GPS), which is scored according to C‐reactive protein (CRP) and albumin levels. Recently, Kaibori et al.^16^ developed a new prognostic score, named the neo‐GPS, that combines the serum CRP level and ALBI grade for prediction of prognosis in patients with HCC who have received surgical resection.

In this study, we researched whether the neo‐GPS at the beginning of treatment predicts outcomes in Atez/Bev‐treated patients with HCC. Additionally, we compared the ability of the GPS and neo‐GPS for prediction of survivals in these HCC patients.

## 2 MATERIALS AND METHODS

### 2.1 Patient selection

This retrospective study was conducted in accordance with the Guidelines for Clinical Research issued by the Japanese Ministry of Health and Welfare, and examined patient record data. The protocol of this study was approved by the institutional ethics committee of Ehime Prefectural Central Hospital (UMIN000043219) (IRB No. 30–66). All procedures were performed in accordance with the Declaration of Helsinki. Written informed consent was obtained from all subjects.

Between September 2020 and March 2022, a total of 467 patients received Atez/Bev therapy for advanced HCC at 22 hospital groups and institutions in Japan. Of these, 8 patients whose last follow‐up date was unknown and 38 patients for whom CRP data were not available at the start of follow‐up were excluded. Consequently, 421 patients were enrolled in the study.

The etiology of HCC in this study was determined to be hepatitis C virus in patients with positive for hepatitis C virus antibodies, and hepatitis B virus in patients with positive for hepatitis B virus surface antigen.

The Child–Pugh score^17^ and ALBI score/grade^9^ were used to assess hepatic reserve function. The neutrophil‐to‐lymphocyte ratio (NLR) was confirmed by dividing the count of neutrophil by the count of lymphocyte.^18^

The start date of Atez/Bev administration was identified as the start date of follow‐up for this study. The end date of follow‐up of the study was identified as the date of last visit for patients who were alive, and the date of death for patients who died during the observation period.

### 2.2 GPS and neo‐GPS

Patients who had an elevated serum CRP level (>1.0 mg/dl) and low albumin level (<3.5 g/dl) were defined as having a GPS of 2. Patients with only one of these outside the standard value had a GPS of 1, and those who had neither had a GPS of 0.^10^, ^11^, ^12^, ^13^, ^14^, ^15^

Patients who had a serum CRP level of >1.0 mg/dl and ALBI grade 2 or 3 were defined as having a neo‐GPS of 2. Patients with only one of these outside the standard value had a neo‐GPS of 1, and those who had neither had a neo‐GPS of 0.^16^

### 2.3 HCC diagnosis and treatment

HCC was diagnosed by dynamic computed tomography (CT), dynamic magnetic resonance imaging, contrast‐enhanced ultrasonography with perflubutane, or histopathologic findings in addition to elevated α‐fetoprotein levels.^19^, ^20^ HCC stage was defined based on the Barcelona Clinic Liver Cancer (BCLC) classification system.^21^

Treatment of Atez/Bev was indicated for unresectable HCC. However, even with BCLC stage A, when surgical resection was not possible due to cardiopulmonary function or when local ablation therapy was difficult due to the surrounding organs of the liver or intrahepatic vessels. In addition, in patients with BCLC stage D, the attending physician in each hospital considered the risks and benefits of Atez/Bev treatment for these patients.

The optimal treatment for each patient's HCC was determined by discussions among oncologists, hepatologists, surgeons, and radiologists at each hospital in accordance with the Japanese practice guidelines for HCC.^22^, ^23^

### 2.4 Atez/Bev treatment and adverse event evaluation

Patients intravenously received 1200 mg of Atez followed by 15 mg/kg of Bev on the same days every 3 weeks.^3^ This treatment was discontinued in the event of clinical tumor progression or unacceptable or serious adverse events.

Adverse events were evaluated according to the National Cancer Institute Common Terminology Criteria for Adverse Events, version 5.0.^24^ In an adverse event developed, it was determined whether one or both drugs should be reduced or discontinued based on the guidelines for this therapy provided by the manufacturer. Immune‐related adverse events were confirmed by each attending physician. If treatment of Atez/Bev was discontinued, the attending physician decided to introduce another treatment regimen in accordance with the Japanese practice guidelines for the treatment of HCC.^22^, ^23^

### 2.5 Evaluation of therapeutic response

The Response Evaluation Criteria in Solid Tumors (RECIST), ver. 1.1,^25^ was used to evaluate treatment response [complete response (CR), partial response (PR), stable disease (SD), progressive disease (PD)]. Whenever possible, initial assessment of the treatment response was performed by dynamic CT results obtained approximately 6 weeks after the introduction of Atez/Bev therapy, then additional examinations of dynamic CT were performed as needed depending on the patient's condition of a disease, sometimes even within 6 weeks after the initial evaluation. Beyond 6 weeks, examinations of dynamic CT were performed every 6 weeks and then every 9–12 weeks after the 6 months from Atez/Bev therapy started.

### 2.6 Statistical analysis

Cumulative progression‐free survival and overall survival was evaluated by the Kaplan–Meier method, and differences were evaluated with the log‐rank test with Bonferroni correction. Multivariate Cox proportional hazards modeling analysis was carried out to assess factors associated with progression‐free survival and overall survival. In this study, we defined two models for the factors included in the multivariate analysis. Both models included age, sex, HCC etiology (viral/non‐viral), Atez/Bev therapy type (first line/other), α‐fetoprotein, NLR, and BCLC stage (≤B/≥C); model 1 also contained the GPS, while model 2 instead contained the neo‐GPS. According to previous reports, cutoff values for consecutive clinical factors were defined as follows: age, 75 years; α‐fetoprotein, 100 ng/ml; and NLR, 3.0.^2^, ^18^ The Akaike information criterion (AIC)^26^ was used to analyze the discriminative ability of the scoring model, and the c‐index was used to assess its prognostic ability. Numerical variables are presented as medians (interquartile range).

All of the reported p‐values were two‐sided. If it was less than 0.05, a result was considered significant. All statistical analyses were conducted using EZR V. 1.55 (Saitama Medical Center, Jichi Medical University, Saitama, Japan).^27^

## 3 RESULTS

### 3.1 Patient characteristics

The baseline characteristics of the 421 study patients are shown in Table 1. There were 340 (80.8%) males and 81 (19.2%) females, with a median age of 74.0 years (68.0–79.0). The numbers of patients with a GPS of 0, 1, or 2 were 239 (56.8%), 130 (30.9%), and 52 (12.4%), respectively, and the numbers with a neo‐GPS of 0, 1, or 2 were 132 (31.4%), 212 (50.4%), and 77 (18.3%), respectively. There were 4, 55, and 73 patients with BCLC stage of ≤A, B, and ≥ C in neo‐GS of 0, 14, 79, and 119 those of neo‐GPS of 1, and 3, 20, and 54 those of neo‐GPS of 2, respectively (*p* = 0.097). The median follow‐up duration was 8.7 (5.0–13.2) months. There were 115 patients who died during the follow‐up period.

**TABLE 1:** Patient characteristics (*n* = 421)

Age^a^ (years)	74.0 (68.0–79.0)
Sex (female/male)	81/340
ECOG‐PS (0/1/≥2)	336/71/14
Body mass index (kg/m^2^)	22.9 (20.8–25.6)
Etiology of HCC (hepatitis B/C/B + C/non‐B, non‐C)	68/144/1/208
Albumin (g/dl)^a^	3.7 (3.3–4.1)
Total bilirubin (mg/dl)^a^	0.8 (0.6–1.0)
CRP (mg/dl)^a^	0.30 (0.10–0.79)
Platelet count (×10^3^/m^3^)^a^	13.9 (10.6–18.9)
Neutrophils (×10^3^/m^3^)^a^	2920 (2158–3927)
Lymphocytes (×10^3^/m^3^)^a^	1130 (790–1521)
Prothrombin time (%)^a^	90 (82–100)
α‐fetoprotein level (ng/ml)^a^	42.5 (6.7–581.0)
Child–Pugh score (5/6/≥7)	246/144/31
ALBI grade (1/2/3)	148/269/4
BCLC stage (≤A/B/≥C)	21/154/246
NLR^a^	2.58 (1.84–3.66)
GPS (0/1/2)	239/130/52
Neo‐GPS (0/1/2)	132/212/77
Atez/Bev therapy type (first line/other)	259/162
Follow‐up duration^a^ (months)	8.7 (5.0–13.2)

Abbreviations: ALBI, albumin–bilirubin; Atez/Bev, atezolizumab plus bevacizumab; BCLC, Barcelona Clinic Liver Cancer; CRP, C‐reactive protein; ECOG‐PS, Eastern Cooperative Oncology Group Performance Status; GPS, Glasgow prognostic score; HCC, hepatocellular carcinoma; NLR, neutrophil‐to‐lymphocyte ratio.

^a^ Data expressed as medians (interquartile range).

### 3.2 Overall and progression‐free survival

**FIGURE 1:**
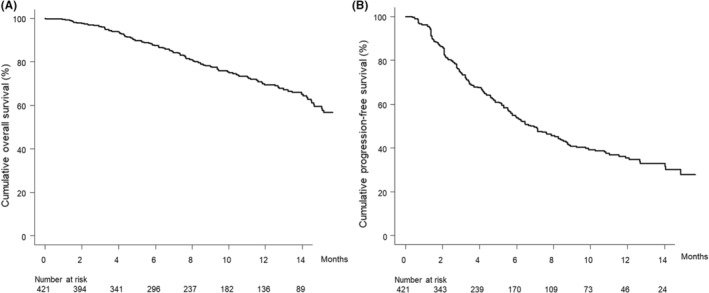
(A) Cumulative overall survival curve. The cumulative overall survival rates at 3, 6, and 12 months are 96.3%, 87.4%, and 69.4%, respectively. (B) Cumulative progression‐free survival curve. The cumulative progression‐free survival rates at 3, 6, and 12 months were 74.2%, 53.9%, and 35.5%, respectively.

Figure 1A shows the curve for overall survival in this cohort. The median survival time was 17.8 (95% confidence interval [CI], 15.1–not available [NA]) months. Figure 1B shows the curve for progression‐free survival in this cohort. The median progression‐free survival time was 6.7 (95% CI, 5.8–8.3) months.

### 3.3 Clinical factors associated with overall and progression‐free survival

Clinical factors associated with overall survival in the univariate analysis are listed in Table 2. The variables such as α‐fetoprotein, BCLC stage, NLR, GPS of 1 and 2, and neo‐GPS of 1 and 2 were statistically significant.

**TABLE 2:** Multivariate analysis of overall survival

	Univariate analysis			Multivariate analysis					
				Model 1			Model 2		
	HR	95% CI	*p* value	HR	95% CI	*p* value	HR	95% CI	*p* value
**Age (years)**									
<75 (*n* = 233)	1	0.989–2.061	0.057	1	1.013–2.197	0.043	1	0.995–2.137	0.053
≥75 (*n* = 188)	1.428			1.492			1.458		
**Sex**									
Female (*n* = 81)	1	0.588–1.467	0.750	1	0.523–1.352	0.474	1	0.599–1.535	0.860
Male (*n* = 340)	0.928			0.841			0.959		
**Etiology**									
Viral (*n* = 213)	1	0.790–1.643	0.848	1	0.669–1.432	0.912	1	0.593–1.282	0.486
Non‐viral (*n* = 208)	1.140			0.979			0.872		
**α‐fetoprotein (ng/mL)**									
<100 (*n* = 247)	1	1.602–3.373	<0.001	1	1.200–2.671	0.004	1	1.263–2.779	0.002
≥100 (*n* = 174)	2.325			1.790			1.873		
**BCLC stage**									
≤B (*n* = 175)	1	1.100–2.459	0.015	1	0.751–1.815	0.492	1	0.766–1.848	0.440
≥C (*n* = 246)	1.645			1.167			1.190		
**Atez/Bev therapy type**									
First line (*n* = 259)	1	0.811–1.698	0.396	1	0.642–1.380	0.755	1	0.615–1.340	0.626
Other (*n* = 162)	1.173			0.941			0.908		
**NLR**									
<3 (*n* = 247)	1	1.438–3.027	<0.001	1	1.055–2.358	0.026	1	1.149–2.548	0.008
≥3 (*n* = 163)	2.087			1.578			1.711		
**GPS**									
0 (*n* = 239)	1			1					
1 (*n* = 130)	2.087	1.370–3.180	<0.001	1.711	1.106–2.646	0.016			
2 (*n* = 52)	4.957	3.066–8.014	<0.001	4.643	2.778–7.762	<0.001			
**Neo‐GPS**									
0 (*n* = 132)	1						1		
1 (*n* = 212)	3.335	1.898–5.861	<0.001				3.038	1.715–5.383	<0.001
2 (*n* = 77)	6.249	3.414–11.440	<0.001				5.312	2.853–9.890	<0.001

Abbreviations: Atez/Bev, atezolizumab plus bevacizumab; BCLC, Barcelona Clinic Liver Cancer; GPS, Glasgow prognostic score; LR, neutrophil‐to‐lymphocyte ratio.

Multivariate analysis based on model 1 showed that age ≥ 75 years (hazard ratio (HR), 1.492; 95% CI, 1.013–2.197; *p* = 0.042), α‐fetoprotein level ≥ 100 ng/ml (HR, 1.790; 95% CI, 1.200–2.671; *p* = 0.004), NLR ≥3 (HR, 1.578; 95% CI, 1.055–2.358; *p* = 0.026), GPS of 1 (HR, 1.711; 95% CI, 1.106–2.646; *p* = 0.016), and GPS of 2 (HR, 4.643; 95% CI, 2.778–7.762; *p* < 0.001) were independently associated with overall survival (Table 2).

Multivariate analysis based on model 2 showed that an α‐fetoprotein level ≥ 100 ng/ml (HR, 1.873; 95% CI, 1.263–2.779; *p* = 0.002), NLR ≥3 (HR, 1.711; 95% CI, 1.149–2.548; *p* = 0.008), neo‐GPS of 1 (HR, 3.038; 95% CI, 1.715–5.383; *p* < 0.001), and neo‐GPS of 2 (HR, 5.312; 95% CI, 2.853–9.890; *p* < 0.001) were independently associated with overall survival (Table 2).

Clinical factors associated with progression‐free survival in the univariate analysis are listed in Table 3. The f variables such as α‐fetoprotein, NLR, GPS of 1 and 2, and neo‐GPS of 2 were statistically significant.

**TABLE 3:** Multivariate analysis of progression‐free survival

	Univariate analysis			Multivariate analysis					
				Model 1			Model 2		
	HR	95% CI	*p* value	HR	95% CI	*p* value	HR	95% CI	*p* value
**Age (years)**									
<75 (*n* = 233)	1	0.837–1.421	0.522	1	0.850–1.469	0.427	1	0.843–1.454	0.466
≥75 (*n* = 188)	1.090			1.117			1.107		
**Sex**									
Female (*n* = 81)	1	0.898–1.796	0.176	1	0.923–1.895	0.128	1	0.954–1.949	0.089
Male (*n* = 340)	1.270			1.323			1.363		
**Etiology**									
Viral (*n* = 213)	1	0.834–1.411	0.543	1	0.771–1.330	0.928	1	0.743–1.288	0.873
Non‐viral (*n* = 208)	1.085			1.013			0.978		
**α‐fetoprotein level (ng/mL)**									
<100 (*n* = 247)	1	1.359–2.302	<0.001	1	1.264–2.227	<0.001	1	1.300–2.282	<0.001
≥100 (*n* = 174)	1.769			1.678			1.723		
**BCLC stage**									
≤B (*n* = 175)	1	0.944–1.619		1	0.702–1.251	0.659	1	0.715–1.277	0.758
≥C (*n* = 246)	1.236			0.937			0.955		
**Atez/Bev therapy type**									
First line (*n* = 259)	1	0.953–1.617	0.109	1	0.842–1.451	0.472	1	0.836–1.444	0.500
Other (*n* = 162)	1.241			1.105			1.098		
**NLR**									
<3 (*n* = 247)	1	1.181–2.016	0.001	1	1.005–1.778	0.046	1	1.034–1.831	0.028
≥3 (*n* = 163)	1.543			1.337			1.376		
**GPS**									
0 (*n* = 239)	1			1					
1 (*n* = 130)	1.495	1.118–1.999	0.007	1.361	1.008–1.837	0.044			
2 (*n* = 52)	2.216	1.499–3.276	<0.001	1.777	1.172–2.694	0.007			
**Neo‐GPS**									
0 (*n* = 132)	1						1		
1 (*n* = 212)	1.342	0.983–1.832	0.064				1.292	0.938–1.778	0.116
2 (*n* = 77)	2.091	1.435–3.046	<0.001				1.763	1.180–2.616	0.005

Abbreviations: Atez/Bev, atezolizumab plus bevacizumab; BCLC, Barcelona Clinic Liver Cancer; GPS, Glasgow prognostic score; NLR, neutrophil‐to‐lymphocyte ratio.

Multivariate analysis based on model 1 showed that an α‐fetoprotein level ≥ 100 ng/ml (HR, 1.678; 95% CI, 1.264–2.227; *p* < 0.001), NLR ≥3 (HR, 1.337; 95% CI, 1.005–1.778; *p* = 0.046), GPS of 1 (HR, 1.361; 95% CI, 1.008–1.837; *p* = 0.044), and GPS of 2 (HR, 1.777; 95% CI, 1.172–2.694; *p* = 0.007) were independently associated with progression‐free survival (Table 3).

Multivariate analysis based on model 2 showed that an α‐fetoprotein level ≥ 100 ng/ml (HR, 1.723; 95% CI, 1.300–2.282; *p* < 0.001), NLR ≥3 (HR, 1.376; 95% CI, 1.034–1.831; *p* = 0.028), and neo‐GPS of 2 (HR, 1.763; 95% CI, 1.188–2.616; *p* = 0.005) were independently associated with progression‐free survival (Table 3).

### 3.4 Overall survival according to the GPS and neo‐GPS

**FIGURE 2:**
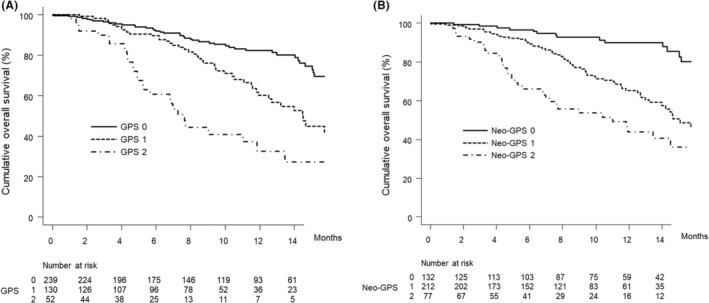
(A) Cumulative overall survival curves by GPS score. The cumulative overall survival rates of patients with a GPS score of 0 at 3, 6, and 12 months are 96.5%, 92.0%, and 82.3%, respectively (solid line). In patients with a GPS score of 1, the cumulative overall survival rates are 98.4%, 89.7%, and 60.2% at 3, 6, and 12 months, respectively (dotted line). The cumulative overall survival rates of patients with a GPS score of 2 are 89.9%, 60.7%, and 32.6% at 3, 6, and 12 months, respectively (dashed‐dotted line) (*p* < 0.001, log‐rank test). The cumulative overall survival rates differed significantly between the GPS 0 group and GPS 1 group (*p* < 0.001), GPS 0 group and GPS 2 group (*p* < 0.001), and GPS 1 group and GPS 2 group (*p* = 0.004) after Bonferroni correction. The AIC and c‐index were 1211 and 0.652, respectively. (B) Cumulative overall survival curves by neo‐GPS score. The cumulative overall survival rates of patients with a neo‐GPS score of 0 at 3, 6, and 12 months are 98.4%, 96.6%, and 90.0%, respectively (solid line). In patients with a neo‐GPS score of 1, the cumulative overall survival rates are 97.1%, 89.3%, and 65.3% at 3, 6, and 12 months, respectively (dotted line). The cumulative overall survival rates of patients with a neo‐GPS score of 2 are 90.3%, 66.0%, and 43.8% at 3, 6, and 12 months, respectively (dashed‐dotted line) (*p* < 0.001, log‐rank test). The cumulative overall survival rate differed significantly between the neo‐GPS 0 group and neo‐GPS 1 group (*p* < 0.001), neo‐GPS 0 group and neo‐GPS 2 group (*p* < 0.001), and neo‐GPS 1 group and neo‐GPS 2 group (*p* = 0.006) after Bonferroni correction. The AIC and c‐index were 1207 and 0.677, respectively. GPS, Glasgow prognostic score.

The median overall survival times in patients with a GPS of 0, 1, or 2 were NA months (95% CI: 17.5–NA), 14.5 months (95% CI: 11.9–NA), and 7.6 months (95% CI: 5.2–11.8), respectively (*p* < 0.001) (Figure 2A), while those in patients with a neo‐GPS of 0, 1, or 2 were NA (95% CI: NA–NA), 15.1 months (95% CI: 13.2–NA), and 11.0 months (95% CI: 7.0–NA), respectively (*p* < 0.001) (Figure 2B). The neo‐GPS, compared with the GPS, had a lower AIC (1207 vs. 1211, respectively) and higher c‐index (0.677 vs. 0.652, respectively) in terms of overall survival.

### 3.5 Treatment response

The radiological best treatment response rates for CR, PR, SD, and PD were 3.1%, 23.7%, 53.4%, and 19.8%, respectively (Table 4). The overall response rate was 26.7%, and the disease control rate (DCR) was 80.2% (Table 4). Treatment responses by neo‐GPS are also listed in Table 4. DCRs differed significantly by neo‐GPS score.

**TABLE 4:** Therapeutic response

	Overall				
	(*n* = 421)	Neo‐GPS 0 (*n* = 132)	Neo‐GPS 1 (*n* = 212)	Neo‐GPS 2 (*n* = 77)	*p* value
CR	12 (3.1%)	5 (4.0%)	7 (3.6%)	0 (0.0%)	0.081
PR	93 (23.7%)	27 (21.6%)	47 (24.1%)	19 (26.0%)	
SD	210 (53.4%)	75 (60.0%)	103 (52.8%)	32 (43.8%)	
PD	78 (19.8%)	18 (14.4%)	38 (19.5%)	22 (30.1%)	
Not evaluated	28	7	17	4	
ORR	26.7%	25.6%	27.7%	26.0%	0.908
DCR	80.2%	85.6%	80.5%	69.9%	0.027

Abbreviations: CR, complete response; DCR; disease control rate; GPS, Glasgow prognostic score; ORR, overall response rate; PD, progressive disease; PR, partial response; SD, stable disease.

In this study, there were 28 patients who either did not reach the follow‐up period before the first treatment response evaluation or stopped treatment with Atez/Bev before the first therapeutic response evaluation.

### 3.6 Adverse events

Table 5 lists the treatment‐related adverse events by Atez/Bev in this study, both overall and by neo‐GPS. Immune‐related liver injury adverse event of any grade and grade ≥3, decreased appetite of any grade, and fever of any grade differed significantly by neo‐GPS.

**TABLE 5:** Adverse events

	Overall (*n* = 421)	Neo‐GPS 0 (*n* = 132)	Neo‐GPS 1 (*n* = 212)	Neo‐GPS 2 (*n* = 77)	*p* value
Immune‐related liver injury					
Any grade	51 (12.1%)	9 (6.8%)	26 (12.3%)	16 (20.8%)	0.012
Grade ≥3	13 (3.1%)	1 (0.8%)	6 (2.8%)	6 (7.8%)	0.017
Immune‐related thyroid dysfunction					
Any grade	25 (5.9%)	9 (6.8%)	14 (6.6%)	2 (2.6%)	0.389
Grade ≥3	4 (1.0%)	1 (0.8%)	3 (1.4%)	0 (0.0%)	0.528
Proteinuria					
Any grade	140 (33.3%)	35 (26.5%)	74 (34.9%)	31 (40.3%)	0.097
Grade ≥3	41 (9.7%)	9 (6.8%)	26 (12.3%)	6 (7.8%)	0.207
Fatigue					
Any grade	101 (24.0%)	26 (19.7%)	53 (25.0%)	22 (28.6%)	0.311
Grade ≥3	7 (1.7%)	4 (3.0%)	2 (0.9%)	1 (1.3%)	0.326
Decreased appetite					
Any grade	94 (22.3%)	18 (13.6%)	53 (25.0%)	23 (29.9%)	0.010
Grade ≥3	13 (3.1%)	2 (1.5%)	9 (4.2%)	2 (2.6%)	0.350
Hypertension					
Any grade	69 (16.4%)	23 (17.4%)	35 (16.5%)	11 (14.3%)	0.838
Grade ≥3	18 (4.3%)	5 (3.8%)	11 (5.2%)	2 (2.6%)	0.595
Fever					
Any grade	30 (7.1%)	6 (4.5%)	13 (6.1%)	11 (14.3%)	0.022
Grade ≥3	6 (1.4%)	1 (0.8%)	4 (1.9%)	1 (1.3%)	0.688
Other					
Any grade	201 (47.7%)	54 (40.9%)	108 (50.9%)	39 (50.6%)	0.165
Grade ≥3	61 (14.5%)	12 (9.1%)	35 (16.5%)	14 (18.2%)	0.098

Abbreviations: GPS, Glasgow prognostic score.

### 3.7 Subgroup analysis of the neo‐GPS in terms of overall survival

**FIGURE 3:**
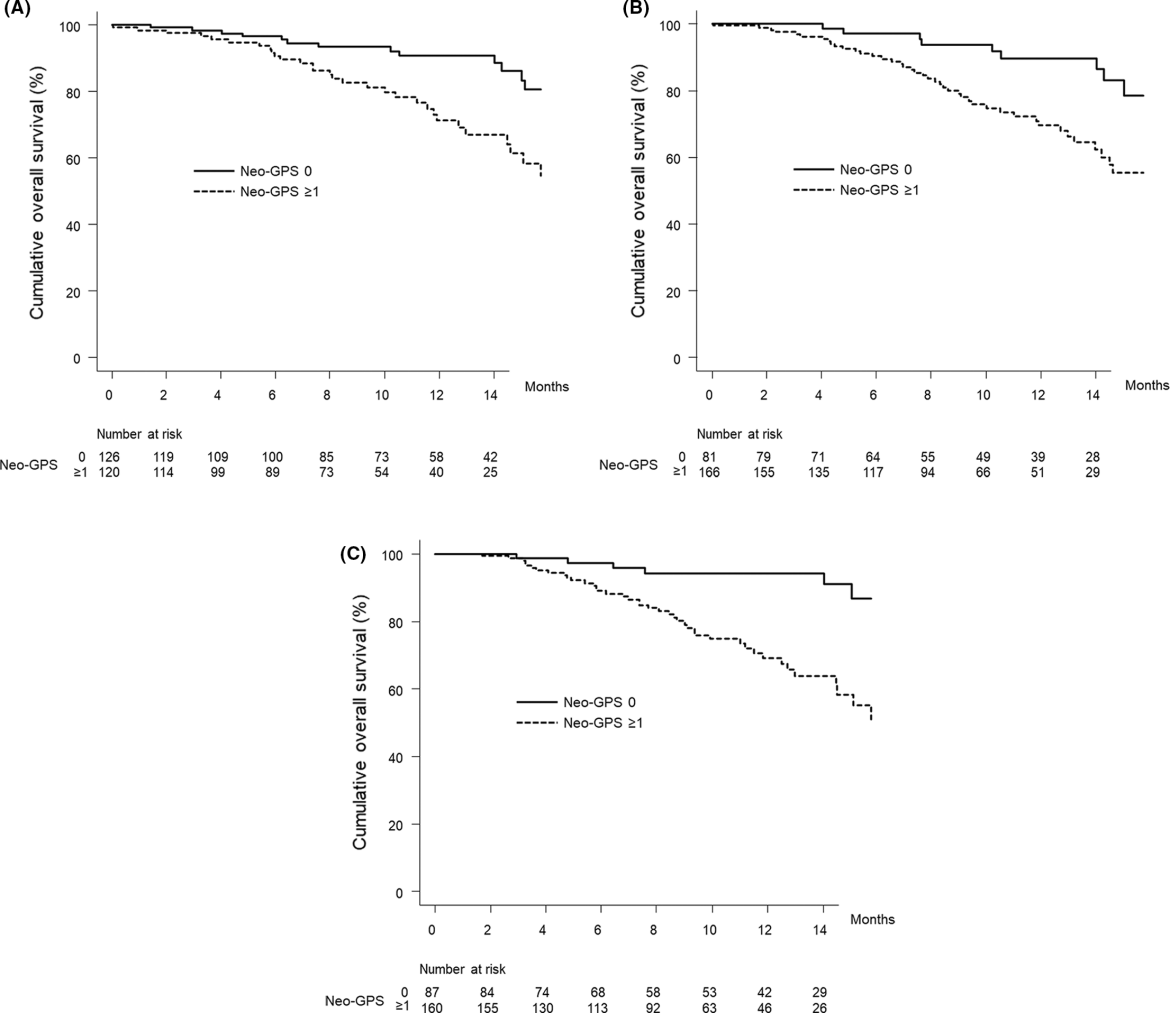
Cumulative overall survival curves by neo‐GPS score in patient subgroups. (A) Patients with a Child–Pugh score of 5. The cumulative overall survival rates of patients with a neo‐GPS score of 0 at 3, 6, and 12 months are 98.3%, 96.5%, and 90.7%, respectively (solid line). In patients with a neo‐GPS score of ≥1, the cumulative overall survival rates are 97.5%, 90.5%, and 71.2% at 3, 6, and 12 months, respectively (dotted line) (*p* = 0.001, log‐rank test). (B) Patients with NLR <3. The cumulative overall survival rates of patients with a neo‐GPS score of 0 at 3, 6, and 12 months are 100.0%, 97.1%, and 89.6%, respectively (solid line). In patients with a neo‐GPS score of ≥1, the cumulative overall survival rates are 97.5%, 90.2%, and 69.6% at 3, 6, and 12 months, respectively (dotted line) (*p* = 0.001, log‐rank test). (C) Patients with α‐fetoprotein < 100 ng/ml. The cumulative overall survival rates of patients with a neo‐GPS score of 0 at 3, 6, and 12 months are 98.8%, 97.4%, and 94.3%, respectively (solid line). In patients with a neo‐GPS score of ≥1, the cumulative overall survival rates are 98.7%, 89.0%, and 69.1% at 3, 6, and 12 months, respectively (dotted line) (*p* < 0.001, log‐rank test). GPS, Glasgow prognostic score; NLR, neutrophil‐to‐lymphocyte ratio.

Figure 3A shows the curves for overall survival stratified by neo‐GPS in patients with a Child–Pugh score of 5. The median overall survival was NA (95% CI, NA–NA) months in patients with a neo‐GPS of 0 and 17.8 (95% CI, 14.6–NA) months in those with a neo‐GPS of ≥1 (*p* = 0.001).

Figure 3B shows the curves for overall survival stratified by neo‐GPS in patients with an NLR <3. The median overall survival was NA (95% CI, NA–NA) months in patients with a neo‐GPS of 0 and 17.8 (95% CI, 14.2–NA) months in patients with a neo‐GPS of ≥1 (*p* = 0.001).

Figure 3C shows the curves for overall survival stratified by neo‐GPS in patients with α‐fetoprotein <100 ng/ml. The median overall survival was NA (95% CI, NA–NA) months in patients with a neo‐GPS of 0 and NA (95% CI, 14.5–NA) months in those with a neo‐GPS of ≥1 (*p* < 0.001).

## 4 DISCUSSION

In this Japanese multicenter study, high neo‐GPSs were associated with poor prognosis in patients with HCC who received Atez/Bev therapy, the combination therapy of an immune checkpoint inhibitor (Atez) and an antibody against vascular endothelial growth factor (Bev) that was developed as first‐choice therapy for patients with unresectable HCC. After adjusting for sex, age, HCC etiology, history of systematic therapy, α‐fetoprotein level, NLR, neo‐GPS, and BCLC stage, an α‐fetoprotein level ≥ 100 ng/ml, NLR ≥3, neo‐GPS of 1, and neo‐GPS of 2 were significantly associated with overall survival, while an α‐fetoprotein level ≥ 100 ng/ml, NLR ≥3, and neo‐GPS of 2 were independently associated with progression‐free survival. Additionally, compared to the GPS, which has been previously investigated to be a valuable prognostic tool for HCC patients,^12^, ^13^, ^14^, ^15^ the neo‐GPS had a lower AIC and higher c‐index for overall survival. In other words, the neo‐GPS had better discriminatory capacity and predictive ability for overall survival than the GPS in advanced unresectable HCC patients who received Atez/Bev therapy. These results suggest that the neo‐GPS, which is simple and inexpensive to evaluate, can be used to predict the prognosis in patients with advanced unresectable HCC who received Atez/Bev therapy.

The Child–Pugh classification has been used for decades to evaluate liver reserve function and is often employed to HCC staging systems.^17^, ^21^ However, it is weak because of the subjectivity involved in assessing ascites and encephalopathy, and by the correlation between ascites and serum albumin levels.^17^ Additionally, the system was originally reported for patients with cirrhosis, not HCC. The recently developed ALBI grade, which is calculated by only serum albumin and total bilirubin levels, has been reported to have superior predictive ability in assessing prognosis in patients with HCC compared not only to the Child–Pugh classification system^28^, ^29^ but also to the liver damage classification.^30^ Additionally, the ALBI score/grade was developed based on an extensive international database of HCC patients.^9^

Systematic inflammation responses are known to be associated with malignant disease, and some mechanisms of this phenomenon have been clarified to confirm this relationship. For instance, cancer necrosis and hypoxia or damage of local tissue may activate an inflammatory reaction; invasion or cancer growth may cause inflammation of its tissues; and malignant disease cells, malignant cancer‐related leukocytes, or both may influence the increasing of inflammatory cytokines such as factor of neoplasm necrosis, vascular endothelial growth factor, interleukin (IL)‐1, IL‐6, and IL‐8. These inflammatory cytokines and chemokines may promote malignant tumor growth, metastasis, invasion, angiogenesis, and resistance to cytotoxic drugs, and may also subvert the host immune response.^31^, ^32^, ^33^ Among the simple clinical biomarkers of inflammatory responses, CRP has been used as an acute‐phase substance synthesized in hepatocytes and influenced by proinflammatory cytokines such as IL‐6.^34^ Additionally, CRP can be quickly measured at low cost in most clinics and hospitals.

The GPS has been reported as a prognostic factor in patients with digestive system malignancies, including HCC.^10^, ^11^, ^12^, ^13^, ^14^, ^15^ However, the only GPS component associated with nutritional status or liver function is the serum albumin level. Therefore, Kaibori et al.^16^ developed the neo‐GPS, which retains CRP but replaces albumin level with ALBI grade, and reported its usefulness in predicting prognosis in surgically treated patients with HCC. In an analysis of overall survival, they found that the neo‐GPS, in comparison to the GPS, had a lower AIC (1,554 vs. 1,562, respectively) and higher c‐index (0.611 vs. 0.556, respectively).^16^ In addition, regarding complications associated with surgical resection, they found that patients with a high neo‐GPS score (≥1) had a greater rate of high Clavien–Dindo classification (≥3)^35^ compared to those with a high GPS (≥1) (65.1% vs. 32.5%, respectively).^16^ Therefore, it is considered that the neo‐GPS, which combines serum CRP and the ALBI grade, is a reasonable biomarker in HCC patients.^16^, ^36^

In the present study, we demonstrated that in terms of overall survival of patients with advanced HCC who were treated with Atez/Bev, the neo‐GPS, compared with the GPS, was associated with a lower AIC (1,207 vs. 1,211, respectively) and higher c‐index (0.677 vs. 0.652, respectively). In addition, we found that in a subgroup of patients who were found by multivariate analysis to have a good prognosis (NLR <3 or α‐fetoprotein level < 100 ng/mL), those with a high neo‐GPS (≥1) had a significantly poorer prognosis than those with a low neo‐GPS. Furthermore, we showed that the neo‐GPS was able to stratify overall survival even in a subgroup of patients with good liver function reserve, that is, a Child–Pugh score of 5, who were considered to have the best prognosis. Regarding treatment‐related adverse events, higher neo‐GPS was significantly associated with decreased appetite of any grade in more than 20% of study patients.

The advantage of this study is that it clarified that the newly developed neo‐GPS can be a prognostic marker even for patients treated with Atez/Bev, the first‐line systemic therapy for unresectable HCC, although the original study of this score was conducted only in patients who received surgical resection. Various investigations have shown that hepatic function is associated with prognosis in patients treated with systemic therapy, however neo‐GPS which is a combination of ALBI grade and CRP, an inflammatory marker related to the progression of cancer, is thought to have the utility in comprehensively monitoring the clinical course of HCC. This study was conducted only for combination of an immune checkpoint inhibitor and a molecular targeted agent, and it has not been sufficiently investigated whether neo‐GPS can be a prognostic marker even in unresectable HCC patients who were treated by tyrosine kinase inhibitors such as lenvatinib or sorafenib. In the future, it is necessary to investigate whether the prognostic ability of this score is common across different systematic therapies in patients with HCC.

The main limitations of this study include the hospital‐based population and retrospective nature. Although this study investigated a large number of advanced unresectable HCC patients who were treated with Atez/Bev from Japanese multiple centers, future prospective studies should include community‐based patients and those recruited on a nationwide basis, as well as a long‐term observation period. This study did not analyze the HCC treatment after Atez/Bev therapy. Because sequential systemic treatment may affect prognosis in patients with unresectable HCC, further investigations that include an evaluation of HCC treatment after Atez/Bev therapy discontinued are also warranted. Unfortunately, the relationship between future treatment development for HCC and neo‐GPS could not be analyzed in this study. Further studies are warranted to confirmed how this score will help in future research and treatment development for HCC.

In conclusion, the neo‐GPS, a new biomarker that is inexpensive and easy to evaluate in clinical practice, can predict prognosis in HCC patients treated with Atez/Bev, including those who are considered to have a good prognosis. Further studies are needed to confirm these findings in other cohorts.

## ETHICS APPROVAL AND PATIENT CONSENT

The study protocol conformed to the ethical guidelines of the Declaration of Helsinki. The study was approved by the institutional ethics review committee of Ehime Prefectural Central Hospital (UMIN000043219) (IRB No. 30–66). Written informed consent was obtained from each patient before study enrollment.

## AUTHOR CONTRIBUTIONS

**Toshifumi Tada:** Conceptualization (lead); data curation (equal); formal analysis (lead); investigation (lead); resources (equal); writing – original draft (equal). **Takashi Kumada:** Conceptualization (equal); data curation (equal); methodology (equal); project administration (equal); supervision (equal); writing – review and editing (equal). **Atsushi Hiraoka:** Conceptualization (equal); data curation (equal); formal analysis (supporting); investigation (supporting); project administration (supporting); validation (equal); writing – review and editing (equal). **Kazuya Kariyama:** Data curation (equal); writing – review and editing (equal). **Joji Tani:** Data curation (equal); writing – review and editing (equal). **Masashi Hirooka:** Data curation (equal); writing – review and editing (equal). **Koichi Takaguchi:** Data curation (equal); writing – review and editing (equal). **Masanori Atsukawa:** Data curation (equal); writing – review and editing (equal). **Shinya Fukunishi:** Data curation (equal); writing – review and editing (equal). **Ei Itobayashi:** Data curation (equal); writing – review and editing (equal). **Kunihiko Tsuji:** Data curation (equal); writing – review and editing (equal). **Kazuto Tajiri:** Data curation (equal); writing – review and editing (equal). **Hironori Ochi:** Data curation (equal); writing – review and editing (equal). **Toru Ishikawa:** Data curation (equal); writing – review and editing (equal). **Satoshi Yasuda:** Data curation (equal); writing – review and editing (equal). **Chikara Ogawa:** Data curation (equal); writing – review and editing (equal). **Hidenori Toyoda:** Data curation (equal); supervision (supporting); writing – review and editing (equal). **Takeshi Hatanaka:** Conceptualization (supporting); data curation (equal); formal analysis (supporting); investigation (supporting); methodology (supporting); project administration (supporting); writing – review and editing (equal). **Takashi Nishimura:** Data curation (equal); writing – review and editing (equal). **Kakizaki Satoru:** Data curation (equal); writing – review and editing (equal). **Kazuhito Kawata:** Data curation (equal); writing – review and editing (equal). **Noritomo Shimada:** Data curation (equal); writing – review and editing (equal). **Fujimasa Tada:** Data curation (equal); writing – review and editing (equal). **Kazuhiro Nouso:** Data curation (equal); writing – review and editing (equal). **Akemi Tsutsui:** Data curation (equal); writing – review and editing (equal). **Hideko Ohama:** Data curation (equal); writing – review and editing (equal). **Asahiro Morishita:** Data curation (equal); writing – review and editing (equal). **Takuya Nagano:** Data curation (equal); writing – review and editing (equal). **Norio Itokawa:** Data curation (equal); writing – review and editing (equal). **Tomomi Okubo:** Data curation (equal); writing – review and editing (equal). **Taeang Arai:** Data curation (equal); writing – review and editing (equal). **Hisashi Kosaka:** Data curation (equal); writing – review and editing (equal). **Michitaka Imai:** Data curation (equal); writing – review and editing (equal). **Atsushi Naganuma:** Data curation (equal); writing – review and editing (equal). **Shinichiro Nakamura:** Data curation (equal); writing – review and editing (equal). **Yohei Koizumi:** Data curation (equal); writing – review and editing (equal). **Masaki Kaibori:** Data curation (equal); writing – review and editing (equal). **Hiroko Iijima:** Data curation (equal); funding acquisition (lead); supervision (supporting). **Yoichi Hiasa:** Data curation (equal); project administration (supporting); supervision (supporting); writing – review and editing (equal).

## FUNDING INFORMATION

This work was supported by JSPS KAKENHI 21 K07902.

## CONFLICT OF INTEREST

Toshifumi Tada: lecture fees from AbbVie and Eisai. Atsushi Hiraoka: lecture fees from Eisai, Bayer, Otsuka, and Eli Lilly. Takashi Kumada: lecture fees from Eisai. Hidenori Toyoda: lecture fees from Eisai, AbbVie, Gilead, Terumo, and Bayer. Takeshi Hatanaka: lecture fees from Eisai. None of the other authors have potential conflicts of interest to declare.

## Data Availability

The datasets are available from the corresponding author on reasonable request.
